# Identification and Expression Analysis of Polygalacturonase Family Members during Peach Fruit Softening

**DOI:** 10.3390/ijms17111933

**Published:** 2016-11-18

**Authors:** Ming Qian, Yike Zhang, Xiangyan Yan, Mingyu Han, Jinjin Li, Fang Li, Furui Li, Dong Zhang, Caiping Zhao

**Affiliations:** College of Horticulture, Northwest A&F University, Yangling 712100, China; m13637099856@163.com (M.Q.); 18821635518@163.com (Y.Z.); xiangyan0209@163.com (X.Y.); hanmy@nwsuaf.edu.cn (M.H.); lijinjin@bigizy.cn (J.L.); fangfang0918@126.com (Fa.L.); 18821635911@163.com (Fu.L.); afant@nwsuaf.edu.cn (D.Z.)

**Keywords:** peach, PG family, fruit, softening, ripening

## Abstract

Polygalacturonase (PG) is an important hydrolytic enzyme involved in pectin degradation during fruit softening. However, the roles of *PG* family members in fruit softening remain unclear. We identified 45 *PpPG* genes in the peach genome which are clustered into six subclasses. *PpPGs* consist of four to nine exons and three to eight introns, and the exon/intron structure is basically conserved in all but subclass E. Only 16 *PpPG* genes were expressed in ripening fruit, and their expression profiles were analyzed during storage in two peach cultivars with different softening characteristics. Eight *PGs* (*PpPG1*, -*10*, -*12*, -*13*, -*15*, -*23*, -*21*, and -*22*) in fast-softening “Qian Jian Bai” (QJB) fruit and three *PGs* (*PpPG15*, -*21*, and -*22*) in slow-softening “Qin Wang” (QW) fruit exhibited softening-associated patterns; which also were affected by ethylene treatment. Our results suggest that the different softening characters in QW and QJB fruit is related to the amount of *PG* members. While keeping relatively lower levels during QW fruit softening, the expression of six *PGs* (*PpPG1*, -*10*, -*12*, -*11*, -*14*, and -*35*) rapidly induced by ethylene. *PpPG24*, -*25* and -*38* may not be involved in softening of peach fruit.

## 1. Introduction

Fruit maturation is a complex and highly coordinated developmental process. In fleshy fruits, the main changes associated with ripening affect color, firmness, taste and flavor, and increase not only palatability, but also susceptibility to physical damage and shortening of storage life [1,2]. Peach is a typical climacteric fruit, and rapid softening and short shelf life after harvest adversely affect its market value [3]. Therefore, studying the physiological and molecular processes governing the softening of peach would be greatly beneficial in extending its shelf life.

Modification of the cell wall is thought to underlie the changes in fruit firmness and texture [4]. Plant cell wall is a complex reticulate structure, being composed of cellulose, hemicellulose, pectin and structural proteins [5,6]. Among these four macromolecular substances, pectin is the major component in the middle lamella and cell primary wall and can link cells together like “glue” [7,8,9,10]. Thus, systematic disassembly of pectin is critical to some developmental processes in plants, such as organ abscission, fruit ripening, and pod and anther dehiscence [11,12]. The degradation of pectin is catalyzed by hydrolytic enzymes, mainly including polygalacturonase (PG), pectate lyase, pectin methylesterases and β-galactosidase, of which PG has been suggested to play a central role [11,13].

Involvement of PG in fruit softening has been widely reported. In strawberry, silencing of *FaPG1* gene decreases the breakdown of the middle lamella and slows the fruit softening [14]. In pear and banana, fruits softening are positively regulated by *Pc-PG1* and *Pc-PG2*, and *MaPG3* and *MaPG4*, respectively, with a pattern of ethylene-dependent [15,16]. The similar results have also been observed in papaya, guava, capsicum and apple [17,18,19,20]. However, Smith et al. thought *PG* was not essential for tomato fruit softening [21]. Interestingly, peach fruit melting and freestone are also controlled by *Endo-PGs* [22,23,24,25]. In addition, *PG* genes also participate in the organ abscission of lychee, oilseed rape, *Arabidopsis* and tomato [26,27,28]. In *Arabidopsis*, *QUARTET2* (*QRT2*) is involved in pollen grain separation [29], and *ADPG1* and *ADPG2* are related to silique dehiscence and floral organ abscission [30].

PGs belong to one of the largest hydrolase families in plants [31], and the *PG* gene family has been identified in various plant species, including *Arabidopsis* [29], *Oryza sativa* [31], *Populus* [32], *Cucumis sativus* and *Citrullus lanatus* [33]. The expression of 66 *PG* genes of *Arabidopsis* has been tested by RT-PCR in five tissues, flowers, siliques, stems, leaves and roots, but only 43 had PCR products, 40 in flower, 34 in silique and root, 30 in leaf and 31 in stem [31]. In *Populus*, 11 *PG* members are related to flower development and two are related to leaf abscission under salt stress [32]. In *Cucumis sativus*, most *CsPGs* exhibit specific or high expression levels in a particular organ or tissue [33]. These studies have demonstrated that *PG* members are differentially expressed in specific tissues and in response to specific treatments, indicating extensive functional divergence among plant *PG* genes. Although the important roles of PG hydrolase in peach softening were confirmed by many researches [3,4,34], only a small number of *PG* family members have been reported [25,34,35,36]. To clarify which *PG* members are involved in softening of peach fruit, in this study, we identify 45 members of the *PG* family from the whole genomic sequence of peach and analyze the expression profiles of *PpPG* genes during fruit softening in two peach cultivars that have different softening characteristics. We identify a small subset of *PG* genes that display consistent softening-associated expression patterns. We investigate the connection between selected ripening- and softening-related *PG* genes and the mechanism underlying ethylene-dependent ripening.

## 2. Results

### 2.1. Identification of Polygalacturonase (PG) Family Members in Peach

**Figure 1:**
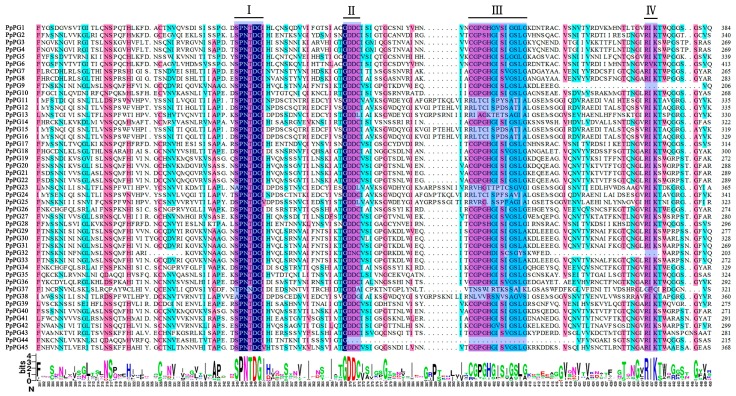
Multiple sequence alignment of peptides of peach PGs. Blue shading and underlining indicates four typical conserved domains of PGs, referred to as domains I, II, III and IV. The consensus sequence is shown by letter logos. Different colors indicate different similarities (black: 100%, Magenta: ≥75%, cyan: ≥50%).

The Hidden Markov Model (HMM) profile of the glycosyl hydrolase family 28 (GH28) domain was used as a query to blast against the NCBI non-redundant protein database and the Peach Genome Database. All reported *Arabidopsis* PG proteins were also used to search against the peach genome database using NCBI tblastn. Then, all candidate *PG* family members in the peach genome database were identified using four highly conserved *PG* domains. Forty-five *PG* genes were identified in peach and named *PpPG1*–*45* according to their chromosomal location (Table 1). Most *PG* family members (32 members) contained the conserved domains I, II, III and IV. *PpPG11*, -*12*, -*13*, -*15*, -*16*, -*23*, -*24*, -*25* and -*38* lacked domain III. *PpPG9* and -*32* did not contain domain IV. *PpPG37* and -*44* lacked domains III and IV, and II and III, respectively (Figure 1).

The open reading frame (ORF) lengths of the 45 *PpPG* family members ranged from 879 to 1866 bp, and the deduced polypeptide sequences ranged in length from 292 to 621 amino acids, with predicted molecular weights (*M*_W_) between 30.66 and 65.91 kDa. The predicted isoelectric points (pIs) of *PpPGs* ranged from 4.88 to 9.53. SignalP 3.0 analysis revealed that *PpPG1*, -*5*, -*6*, -*8*, -*11*, -*12*, -*13*, -*14*, -*16*, -*18*, -*20*, -*21*, -*22*, -*23*, -*24*, -*25*, -*34*, -*36*, -*37*, -*41*, -*42*, -*44* and -*45* contained a signal peptide (Table 1).

### 2.2. Phylogenetic Analysis of PG Family Members in Peach

**Figure 2:**
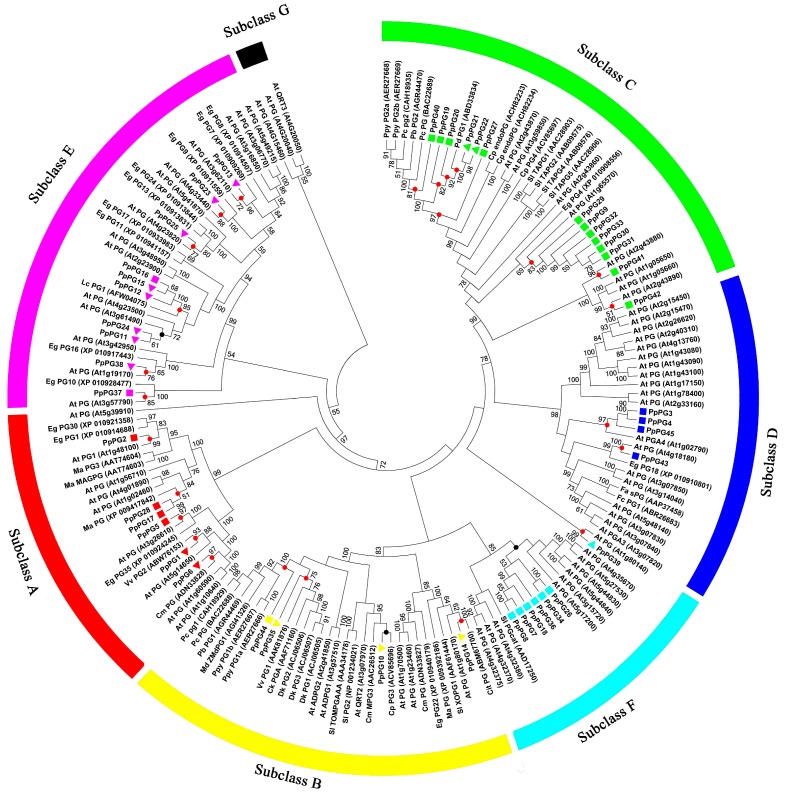
Phylogenetic tree of PGs from *Prunus persica* and other species. Species shown are *Carica papaya* (CpPG), *Citrus sinensis* (CitPG), *Cucumis melo* (CmPG), *Diospyros kaki* (DkPG), *Fragaria chiloensis* (FcPG), *Arabidopsis thaliana* (AtPG), *Fragaria* *× ananassa* (FaPG), *Actinidia chinensis* (CkPGA), *Litchi chinensis* (LcPG), *Malus domestica* (MdPG), *Musa acuminate* (MaPG), *Prunus domestica* subsp. *insititia* (PdPG), *Pyrus communis* (PcPG), *Pyrus bretschneideri* (PbPG), *Pyrus pyrifolia* (PpyPG), *Solanum lycopersicum* (SlPG), *Elaeis guineensis* (EgPG) and *Vitis vinifera* (VvPG). Colored triangles and squares represent *PpPGs* and each color indicates a subclass; triangles indicate *PpPG* genes that are expressed in peach fruit and squares represent *PpPGs* that are not expressed in peach fruit according to results of RNA-seq. Red and black nodes indicate that the bootstrap support for the branches defining the common ancestral PGs is more than 50% and less than 50%, respectively. Bootstrap values lower than 50 are hidden in the unrooted phylogenetic tree.

The phylogenetic tree was created using MEGA 6.0 with full-length PG protein sequences from peach, *Arabidopsis thaliana* and some selected fruit species. The 45 peach PG proteins were clustered into six subclasses (subclasses A to F). Subclass G was *Arabidopsis* *QRT3PG*, which is involved in degradation of the pollen mother cell wall [37], and did not include any peach *PG* members. Most peach *PG* members were included in subclasses C and E, while fewer members were included in subclasses B and D. *PpPG21* and -*22* were similar to *PdPG*; *PpPG12* and *-15* were similar to *LcPG*; and *PpPG1* and -*10* were similar to *VvPG1* and *CmPG1*, respectively (Figure 2). We defined the most likely orthologs between peach and *Arabidopsis*. This suggested that there were at least 25 ancestral *PGs* before the divergence of other species and peach (Figure 2, red circles). Three of these nodes in subclasses of B, E and F, respectively (Figure 2, black circles), however, had relatively low bootstrap support (<50%). *PpPG9*, -*29*, -*30*, -*31*, -*32*, and -*33* in subclass C, and *PpPG7*, -*8*, -*18*, -*26*, -*34*, and -*36* in subclass F, formed two special sub-clades without other species members (Figure 2).

### 2.3. Genome Distribution and Gene Structures of PG Family Members in Peach

**Figure 3:**
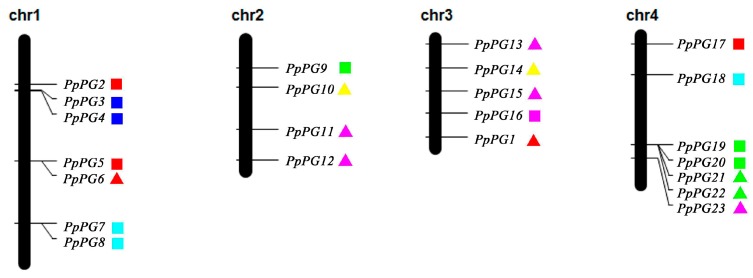

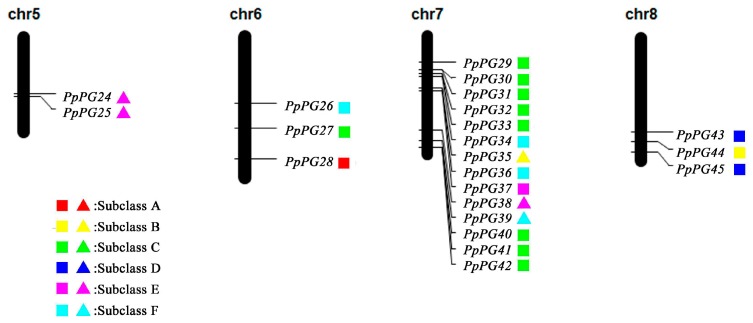
Chromosomal mapping of peach *PG* genes. Each subclass is shown by different color. Triangles, *PpPG* genes expressed in peach fruit; squares, *PpPGs* not expressed in peach fruit, according to results of RNA-seq.

*PG* gene family members are distributed on all peach chromosomes. Chr 7 contains 14 *PpPG* genes; chr 1 and chr 4, and chr 6 and chr 8 contain seven *PpPG* genes and three *PpPG* genes, respectively; chr 2 contains four; chr 3 contains five; and chr 5 contains two. We can also observe tandem duplication sites such as *PpPG8* and -*7* on chr 1, and *PpPG19*, -*20*, -*21* and -*22* on chr 4 (Figure 3).

**Figure 4:**
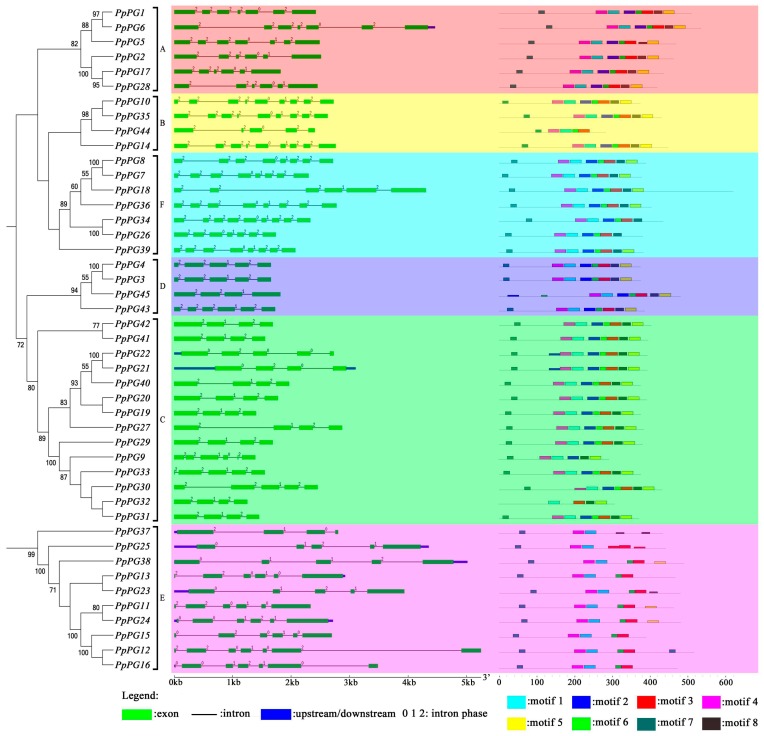
Phylogenetic tree, *PG* gene structures and conserved motif analysis in peach. The left part indicates the phylogenetic tree of *PG* genes in peach. Figures on branches indicate bootstrap percentage values calculated from 1000 replicates with MEGA 6.0, and values lower than 50 are hidden in the phylogenetic tree. The middle part represents intron/exon organization of *PG* genes, and different background colors indicate different subclasses. The right part shows the composition and position of conserved motifs of peach *PG* genes, with eight motifs shown in different colors.

All peach PG protein sequences were used to construct an unrooted phylogenetic tree with MEGA 6.0, and it was consistent with the rooted tree described above (Figure 4). The exon/intron structures and intron phase of peach *PG* genes were analyzed using the online tool GSDS, using their full-length CDS sequences and corresponding genomic DNA sequences. The results showed that *PpPGs* consisted of four to nine exons and three to eight introns, and the members of subclasses A, B and F generally had more exons and introns. In addition, the exon/intron structure is basically conserved in each subclass except for subclass E (Figure 4).

To further investigate conserved motifs in the amino acid sequences of peach *PG* genes, 45 peach PG proteins were aligned using the online tool MEME set to output eight motifs. Motif 1 was found in all 45 peach *PG* members, while motif 4 was absent in *PpPG32*, motif 3 was absent in *PpPG9* and -*37*, and motif 7 was absent in *PpPG32* and -*45*. Some motifs only existed in certain members, such as motif 2, which existed in most *PpPG* members, but not in subclass E. Moreover, most members of subclass E were also missing motif 5 and motif 8. Furthermore, *PpPG44* in subclass B did not have motifs 2, 5 or 8; *PpPG9* in subclass C did not have motifs 3 or 6, and *PpPG32* had only four motifs (Figure 4).

### 2.4. Fruit Firmness, Ethylene Production and PG Activity Change during Softening

**Figure 5:**
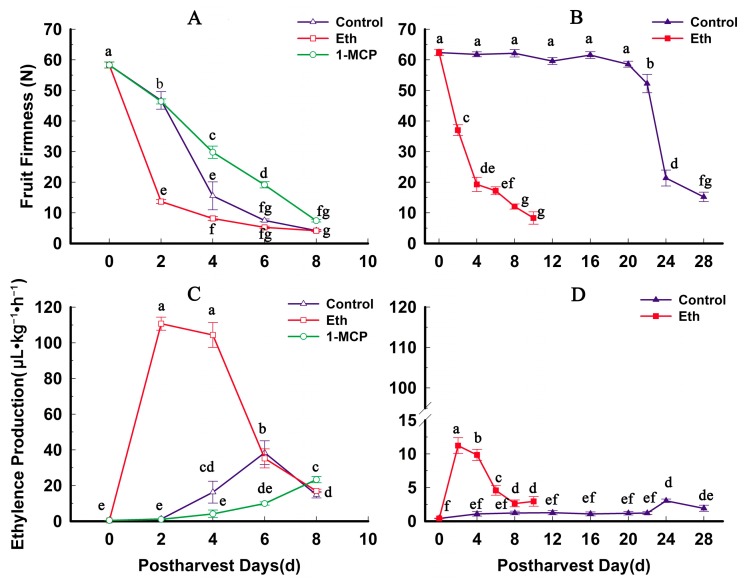
Fruit firmness and ethylene production of control and ethylene-treated fruits during storage in two peach cultivars: (**A**) “Qian Jian Bai” (QJB) firmness; (**B**) “Qin Wang” (QW) firmness; (**C**) QJB ethylene production; and (**D**) QW ethylene production. Data are the means ± standard errors (*n* = 3). Significant differences (*p* < 0.05) between means are indicated by different letters.

The two cultivars have different fruit softening characteristics: QJB fruit softening rapidly during ripening, and QW fruits remain firm for a long time during ripening. The firmness, ethylene production, and PG activity in QJB and QW fruits were measured during fruit storage. For QJB fruit, flesh firmness decreased slowly in the first two days after harvest (DAH), and decreased rapidly from two to four DAH, then declined slowly again (Figure 5A). For QW fruit, flesh firmness remained stable for the first 20 DAH, and decreased slowly from 20 to 22 DAH, then declined dramatically from 22 to 24 DAH (Figure 5B). Ethylene production was very low in QW fruit for the first 22 DAH and then increased markedly from 22 to 24 DAH (Figure 5D). In QJB fruit, ethylene production increased slowly in the first 2 DAH, and increased rapidly from two to six DAH, then decreased considerably (Figure 5C).

**Figure 6:**
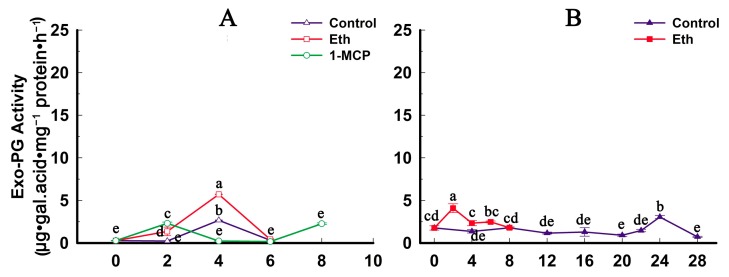

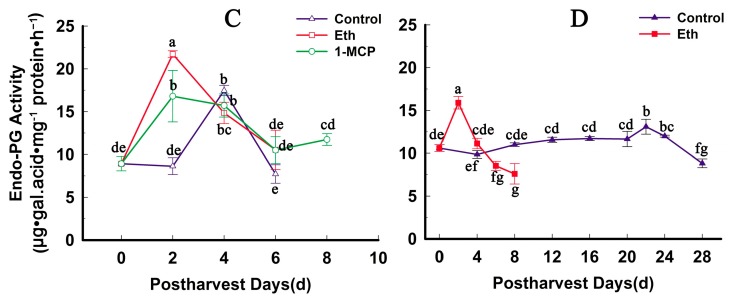
Exo-PG and endo-PG activity of control and ethylene-treated fruits during storage in two peach cultivars: (**A**) QJB exo-PG activity; (**B**) QW exo-PG activity; (**C**) QJB endo-PG activity; and (**D**) QW endo-PG activity. Data are the means ± standard errors (*n* = 3). Significant differences (*p* < 0.05) between means are indicated by different letters.

The activities of exo-PG and endo-PG in the two varieties showed similar trends during storage. In QJB fruit, the activities of exo-PG and endo-PG remained stable for the first two DAH, then increased and reached their maximum activity at 4 DAH, and declined from four to six DAH (Figure 6A,C). In QW fruit, the activities of exo-PG and endo-PG also remained at a stable level in the first 20 DAH, then increased and reached their maximum activity at 24 DAH and 22 DAH, respectively, and then declined (Figure 6B,D). Furthermore, the rate of change of endo-PG activity in QJB fruit was significantly higher than that in QW fruit during storage (Figure 6).

### 2.5. Identification of PG Genes Exhibiting Ripening-Associated Patterns of Expression

We identified the *PG* genes expressed in fruit by transcriptome sequencing of QJB fruit at the ripening stage, and the results showed that among 45 *PpPG* genes, only 16 were expressed in peach fruit (Table S1). These were *PpPG1* and *-6* of subclass A; *PpPG10*, -*35* and -*14* of subclass B; *PpPG21* and -*22* of subclass C; *PpPG25*, -*23*, -*13*, -*15*, -*12*, -*24*, -*11* and -*38* of subclass E; and *PpPG39* of subclass F. Among these 16 genes, nine (*PpPG1*, -*10*, -*12*, -*13*, -*15*, -*21*, -*22*, -*23* and -*25*) were up-regulated during softening, three (*PpPG11*, -*14* and -*39*) were down-regulated, and four (*PpPG6*, -2*4*, -*35* and -*38*) were relatively stable (Table S1).

**Figure 7:**
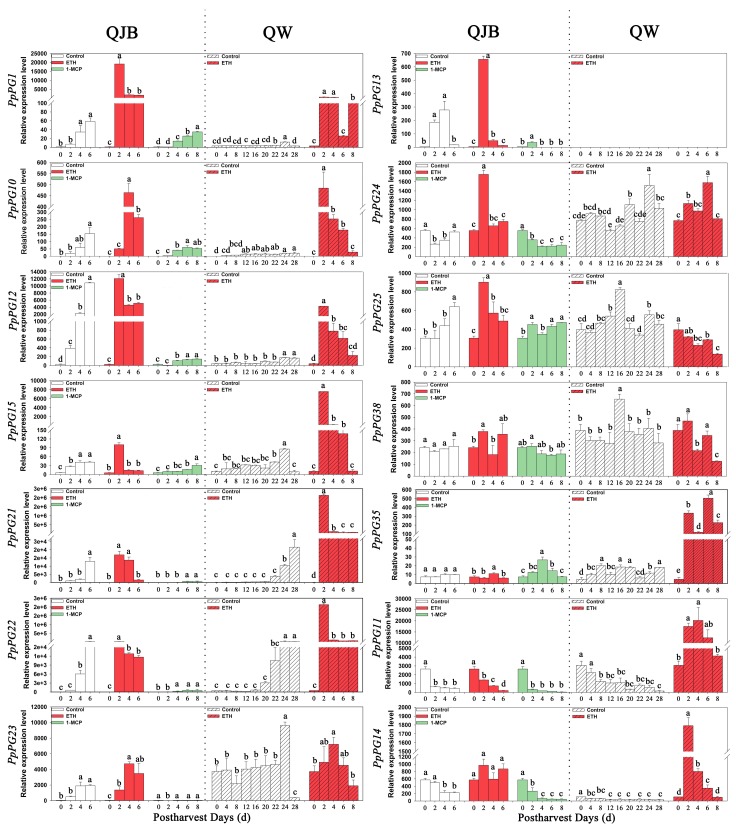
Expression levels of selected *PpPG* genes in control and ethylene-treated fruits during storage in two peach cultivars. 18S served as reference gene. Data are the means ± standard errors (*n* = 3). Significant differences (*p* < 0.05) between means are indicated by different letters.

The transcript accumulation patterns of the 16 *PpPG* genes expressed in peach fruit were assessed in the two varieties by qRT-PCR. The results were consistent with the transcriptome data, and nine *PpPG* genes (*PpPG1*, -*10*, -*12*, -*13*, -*15*, -*21*, -*22*, -*23* and -*25*) showed up-regulation, but some differences existed between the two varieties. For QJB fruit, the expression of *PpPG1*, -*10*, -*12*, -*21* and -*22* was dramatically and continuously increased during the whole storage period, and reached its maximum at the end of storage. *PpPG15*, -*23* and -*13* displayed up-regulation for the first four DAH, and then maintained a stable level or declined rapidly. *PpPG25* expression remained stable in early storage, and then increased continuously (Figure 7).

Compared with QJB, the expression of *PpPG1*, -*10*, -*12* and -*15* in QW fruit showed a lower level of transcript abundance during the whole storage period, with elevated expression levels only from 22 to 24 DAH. *PpPG13* expression was undetectable in QW during the whole storage period. Expression of *PpPG21* and -*22* remained at a very low level for the first 20 DAH, coinciding with the stable firmness. The expression levels subsequently dramatically increased from 20 DAH to 24 or 28 DAH. The expression of *PpPG23* was maintained stably at a high level during the first 22 DAH, increased markedly from 22 to 24 DAH, and then decreased rapidly. *PpPG25* expression remained relatively stable during storage except at 16 DAH (Figure 7).

During storage, the expression of *PpPG11* and *-14* exhibited declining trends in the two varieties (Figure 7). *PpPG24*, -*35* and -*38* also showed similarity in expression abundance and patterns in the two varieties during storage, and expression of these genes remained relatively stable (Figure 7). *PpPG6* and -*39* expression was undetectable in the two varieties.

In addition, transcript abundance among the *PG* genes was significantly different during fruit softening. *PpPG1*, -*12*, -*21*, -*22*, -*23* and -*11* were the most highly expressed during fruit softening; *PpPG10*, -*13*, -*25*, -*14*, -*24* and -*38* were moderately expressed; and *PpPG15* and -*35* showed the lowest level of transcript abundance (Figure 7).

### 2.6. Ethylene and 1-MCP Alter PG Gene Expression

To shed more light on the potential role of *PGs* in fruit ripening, we compared their expression under ethylene and/or 1-MCP treatment during storage. After ethylene treatment, the changes in firmness and ethylene release in the two varieties were very similar. The decrease in fruit firmness was faster than that in the control fruit, especially in the first two DAH. At the same time, after ethylene treatment, the rate of ethylene release increased sharply and peaked at two DAH, with higher peak rates for treated fruit than for controls (Figure 5). Furthermore, in QJB fruit, after ethylene treatment, the expression of eight *PpPG* genes (*PpPG1*, -*12*, -*13*, -*15*, -*21*, -*22*, -*24* and -*25*) also dramatically increased and peaked at two DAH, with higher peak expression for treated fruit than for controls (Figure 7). The expression of *PpPG10* and -*23* was also significantly increased by ethylene treatment, and peaked at four DAH. Compared with the control, ethylene treatment had little effect on the expression of *PpPG11*, *-14*, *-35* and -*38*. For slow-ripening QW fruit, after ethylene treatment, the expression of seven *PpPG* genes (*PpPG1*, -*10*, -*12*, -*14*, -*15*, -*21* and -*22*) increased dramatically and peaked at two DAH, with higher peak expression for treated fruit than for controls (Figure 7). Ethylene treatment also significantly enhanced the expression levels of *PpPG11* and -*35*, and had little effect on the expression of *PpPG13*, -*23*, -*24*, -*25*, and -*38* (Figure 7).

After treatment with 1-MCP, the expression levels of eight *PpPG* genes (*PpPG1*, -*10*, -*12*, -*15*, -*21*, -*22*, -*23* and -*13*) were significantly inhibited in QJB fruit during the whole storage period, with especially obvious declines observed for *PpPG12*, -*21*, -*22* and -*23*. However, expression of *PpPG25*, -*11*, -*14*, -*24*, -*35* and -*38* was little affected by 1-MCP treatment (Figure 7).

## 3. Discussion

### 3.1. PG Family Member Identification and Sequence Analysis

PG is a cell wall hydrolytic enzyme with important roles in many physiological processes, such as fruit ripening, seed germination, cell elongation, organ abscission and pollen tube elongation [4,12,14,20,26,30,38]. *PGs* are encoded by a typical large gene family in plants, and the *PG* gene family has been identified and characterized in some plants such as *Arabidopsis*, *Populus*, rice, *Cucumis sativus* and *Citrullus lanatus* [29,31,32,33]. In this study, we identified 45 *PG* genes in the peach genome, and *Arabidopsis*, *Populus*, *Cucumis sativus* and *Citrullus lanatus* have 68, 75, 53 and 62 *PG* genes, respectively. Peach has fewer *PG* members than the above species, and this may be because peach has not undergone recent whole-genome duplication [39] and the expansion of subclasses is different in different plants.

Most *PG* members from various species contain four conserved domains [33,40]. Domains I and II are likely to compose the catalytic site, domain III can take part in catalytic reactions, and domain IV constitutes a likely candidate for ionic interactions with carboxylate groups present in the substrate [41]. The peach *PG* genes of subclasses A, B, C, D and F contain the four conserved domains and seven to nine motifs, but *PG* members of subclass E lack domain III and contain five or six motifs (Table 1, Figure 4). Other studies in plants have also shown that domain III of *PGs* in subclass E is not conserved [33,40]. Our results showed that most peach *PG* genes of subclass E are expressed in the ripening fruit (Figure 2), which is similar to the results in *Arabidopsis* [31]. In addition, the involvement of *PG* genes of E subclass in fruit abscission was also observed in lychee and *Elaeis guineensis* [26,42] Therefore, most fruit related *PG* genes may belongs to E subclass, which may be related to their conserved functions in fruit abscission and/or ripening. Except for very low expression of *PpPG39*, peach *PG* genes of subclasses D and F are not expressed in ripening fruit, which suggests that these *PG* genes cannot be involved in peach fruit softening during ripening. In addition, *PpPG7*, -*8*, -*18*, -*26*, -*34*, -*36* in subclass F formed a special sub-clades without other species members, and had the low bootstrap support (<50%), which suggesting these PG members may be peach-special. Subclass C contained 14 peach *PG* members; however, only *PpPG21* and -*22* were expressed in the ripening fruit. *PpPG21* and -*22* are 100% similar to PdPG1 (ABD33834), which involved in the regulation of seed maturation and drying, fruit ripening in damson plum [43]. In addition, tomato *TAPG1*, -*2*, -*4*, and -*5* in subclass C were associated with fruit abscission [44,45]. Similarly, Monocot oil palm *EgPG4* gene in subclass C participated in cell wall pectin modifications as well during both mesocarp ripening and fruit shedding [42]. These studies suggested that *PpPG21* and -*22* are probably related to peach fruit ripening and abscission. Two tomato fruit ripening related *PG* genes (AAA34178, and NP_001234021) were classified into Subclass B [46,47], together with other fruit ripening related *PG* genes (such as, *VvPG1* in grape and *ZMdPG1* in apple) [48,49]. Meanwhile, in *Arabidopsis*, *ADPG1* and -*2* in subclass B had been reported to be essential for silique dehiscence, and *ADPG2* and *QRT2* contribute to floral organ abscission [30]. Our results also showed that *PpPG10*, -*14*, and -*35* in group B were expressed in mature fruit, suggesting these PGs may be associated with peach fruit ripening.

### 3.2. Possible Roles of PG Family Members in Fruit Softening during Ripening

The role of PG in fruit softening has been studied extensively, and an increase in PG activity and mRNA levels has been observed during ripening in several fruits [4,11,15,16,17,20,50]. PG hydrolyzes pectin acid along with the its main chain, causing pectin degradation, cell wall dissolution, and ultimately, fruit softening [51]. However, as a multi-gene family, *PG* members were expressed at different stages and in different tissues during the development of plants [31]. Thus far, little is known about the role of *PG* family members in fruit softening. Pan et al. (2015) indicated that *PpPG21* (*ppa006839*) and -*22* (*ppa006857*) were relatively highly expressed in ripening peach fruit, but that their transcripts were almost undetectable in fruit during development [52]. Gu et al. (2016) also demonstrated that *PpPG22* and *-21* are responsible for stone adhesion and melting flesh, respectively [25]. The mRNA levels of *PpPG1* (*ppa018113*) were lower in both the melting type and the stony hard type peach, although they increased slightly after ethylene treatment in the stony hard type peach [36].

Based on the expression profiles of 16 *PpPG* genes during fruit softening and under the treatments of ethylene and/or 1-MCP, a small subset of *PG* genes highly linked to the softening process was identified. In natural ripening fruit of QJB, nine *PGs* (*PpPG1*, -*10*, -*12*, -*13*, -*15*, -*23*, -*21*, -*22* and -*25*) exhibited softening-associated patterns and up-regulated expression during storage. Among these, eight *PGs* (all except *PpPG25*), which displayed dramatic up-regulation after ethylene treatment and significant down-regulation after 1-MCP treatment (Figure 7), emerge as strong candidates to play a role in QJB fruit softening. However, in natural slowly-softening QW fruit, only *PpPG15*, -*21* and -*22* expression showed softening-associated patterns during storage, and also displayed dramatic up-regulation after ethylene treatment (Figure 7), which indicated that *PpPG15*, -*21* and -*22* play a role in QW fruit softening. Thus, as common candidate genes in two varieties, *PpPG15*, -*21* and -*22* may play major roles in peach fruit softening. Moreover, the number of *PG* family members were involved in fruit softening was different in cultivars having different softening characteristics. More *PG* members play a role in fast softening peach varieties, and fewer *PG* members are active in slow-softening peach fruit.

Ethylene has been assigned a major role in the ripening of climacteric fruits, controlling the transcription of softening-related genes [53]. Hiwasa et al. (2003) reported that ethylene is required for *PG* expression even in the late ripening stage [15]. A stony hard type peach fruit characterized by a lack of ethylene production and firm flesh in the mature fruit retained low *PpPG* mRNA expression and endoPG enzyme activity during storage. Ethylene treatment resulted in stony hard type peach fruit softening rapidly and increasing *PpPG* mRNA expression and endo-PG enzyme activity [54,55]. Our results also showed that endo-PG and exo-PG enzyme activity and expression of most *PG* members is induced by exogenous ethylene treatment, but there are differences between two cultivars. *PpPG11*, -*14* and -*35* showed down-regulated or stable expression in naturally ripening QJB and QW fruit during storage, and their expression was not affected by ethylene treatment during QJB storage. However, in QW fruit, the expression of *PpPG11*, -*14* and -*35* increased sharply after ethylene treatment (Figure 7), which suggests that *PpPG11*, -*14* and -*35* do not play a role in softening of QJB fruit, but participate in ethylene-induced ripening and softening in QW fruit.

In addition, the expression of *PpPG1*, -*10* and -*12* remained at a lower level in QW fruit during natural ripening, but increased dramatically throughout the storage period when treated with ethylene. This was accompanied by a rapid increase in ethylene evolution (Figure 7), which suggests that *PpPG1*, -*10* and -*12* rarely affect normal softening of QW peach fruit, but when stimulated with ethylene, their expression levels rapidly increase and accelerate the softening of QW peach fruit. The expression levels of *PpPG24*, -*25* and -*38* were high and stable during the whole storage period, and were hardly affected by ethylene treatment in either QW or QJB variety fruit (Figure 7), which suggests that the expression of these *PGs* is not regulated by ethylene, and not related to peach fruit softening. 

## 4. Materials and Methods

### 4.1. Plant Materials and Treatment

Peach fruit (*Prunus persica* L. Batsch cv. “Qian jian bai” (QJB) and “Qin wang” (QW)) from the Experimental Station of the College of Horticulture, Northwest A&F University in Yangling, Shaanxi, China, were used in this study. In our experiment, flesh texture trait of QW, whose phenotype resembles very much the stony hard type flesh in firmness and crispness, but when fully ripe becomes melting and releases ethylene. This flesh texture trait results in remarkable keeping quality, particularly on the tree, and is rewarding for growers, consumers and researchers [56].

Fruit with no visible defects were harvested when they were commercially mature (fading peel color that is light green, partially red; slightly hard flesh). For QJB, all of the intact fruit were divided randomly into three groups. One group was soaked in 1 g/L ethephon for 15 min at 25 ± 1 °C. The second group was treated with 5 µL/L 1-methylcyclopropene (1-MCP) for 24 h in a closed container, and the third group was soaked in water for 15 min and served as the control group. For QW, fruit were divided into two groups. One group was soaked in 1 g/L ethephon for 15 min at 25 ± 1 °C. The other group was soaked in water and served as the control group. Each group consisted of 150 fruit, which were stored at 25 °C. Samples were taken every other day until the flesh fully softened. All samples were frozen with liquid nitrogen and stored at −80 °C.

### 4.2. RNA Extraction and Reverse Transcription

Total RNA was extracted according to the protocol described by Lester et al. (1994) [35]. RNA integrity and quality were tested using an ultraviolet spectrophotometer (Thermo NanoDrop 2000, Wilmington, DE, USA) and electrophoresis in 1% agarose gels, respectively. Reverse transcription was completed using the PrimeScript RT Reagent Kit with gDNA Eraser (Takara, Beijing, China).

### 4.3. Identification of Peach PG Family Members

All members of the peach *PG* family were identified using two steps. First, hidden Markov model (HMM) profiles of the *PG* domain (Accession No. PF00295) were retrieved from the Pfam database [57]. Second, these domains and identified *Arabidopsis* PG sequences [12] were used as queries to search the GenBank non-redundant protein database and the Peach Genome Database [58]. Then all of the candidate peach *PG* family members were submitted to SMART [59] and Pfam to determine the presence of the domain. Those candidate *PG* family members that contained at least two highly conserved domains (of domains I, II, III, and IV) of *PGs* [60], were considered to be *PGs*.

### 4.4. Multiple Sequence Alignment, Phylogenetic Analysis, Exon/Intron Structure Determination and Chromosome Locations

Multiple sequence alignment was performed and conserved domains were analyzed using DNAMAN 6.0 with default settings. A phylogenetic tree was generated with MEGA 6.0 using the neighbor-joining (NJ) method, and edge support was estimated using 1000 bootstrap replicates. Exon/intron structure was generated with the online software GSDS [61]. Consensus sequence graphs were obtained with the online software WebLogo [62]. Signal peptides were analyzed using SignalP v3.0 [63]. Molecular weights and pIs of the proteins were calculated with the ExPASY Compute pI/*M*_W_ tool [64]. To identify conserved motifs within the *PG* genes, MEME [65] motif analysis was performed. For chromosome locations, data were downloaded from NCBI [66] and genes were mapped to the chromosomes with MapDraw [67].

### 4.5. RNA Deep Sequencing and Library Construction

The QJB fruits from 0, 2, and 4 days after harvest were selected to extracted RNA. The quantity and quality of extracted RNA were tested using using a NanoDrop ND1000 spectro-photometer (Thermo NanoDrop 2000, Wilmington, DE, USA) and electrophoresis in 1% agarose gels, respectively. A ~2-µg total RNA from each sample was used for constructing cDNA libraries and they were sequenced using an Illumina HiSeq ^TM^ 2000 by the Biomarker Biotechnology Corporation (Beijing, China). The experiment was repeated three times. The detailed methods of raw fastq files handling from RNA-seq were carried out according to Xing [68].

### 4.6. Real-Time Quantitative PCR (qPCR) Assays

Real-time quantitative PCR (qPCR) was performed using an iQ5 real-time PCR system (BioRad, Plano, TX, USA). Gene-specific primers were designed using Beacon Designer 8.0 (Table S2). For each sample, 1 µL cDNA, 1 µL of each primer, 2 µL ddH_2_O and 5 µL of 2× SYBR Premix ExTaq II (TaKaRa, Dalian, China) were used for qPCR in a total volume of 10 µL. The qPCR protocol was as specified for the SYBR Premix Ex Taq kit, 1 min at 95 °C, followed by 40 cycles of 15 s at 95 °C, 20 s at annealing temperature and 20 s at 72 °C, followed by 10 s at 95 °C, followed by 40 cycles to construct a melting curve. Peach 18S ribosomal RNA (18S rRNA) was selected as the reference gene. Relative expression level data were analyzed using the 2^−ΔΔ*C*t^ method [69]. Each sample was analyzed in triplicate.

### 4.7. Flesh Firmness, Ethylene Production and Determination of Enzyme Activity

Firmness was measured using the GY-4 firmness meter (Top instrument Co., Ltd., Hangzhou, China) equipped with a 7.9-mm probe. In each group, six fruit were randomly selected and a small piece of epicarp was peeled off from two symmetrical positions on each fruit to attach the probe. Ethylene production was determined as described by Liguori et al. (2004) [70]. Nine fruit were weighed and sealed in a jar for 1 h. An air sample (1 mL) from the jar was analyzed using a gas chromatograph (Trace GC Ultra, Thermo Fisher, New York, NY, USA). The activities of exo-PG and endo-PG were measured according to Cao et al. (2014) [71].

### 4.8. Statistical Analysis

Microsoft Excel 2010 and DPS v7.05 were used for data processing and correlation analysis, and SigmaPlot 10.0 was used to prepare figures.

## 5. Conclusions

In conclusion, a genome-wide analysis of *PG* gene family in peach was performed to reveal gene structure, phylogenetic relationship, and expression profiles during fruit softening. During the normal ripening, eight PGs (*PpPG1*, -*10*, -*12*, -*13*, -*15*, -*23*, -*21*, and -*22*) in fast-softening QJB fruit and three PGs (*PpPG15*, -*21*, and -*22*) in slow-softening QW fruit exhibited softening-associated patterns, which were also affected by ethylene treatment. Our results suggest that the different softening characters in QW and QJB fruit are related to the amount of PG members.

## Figures and Tables

**Table 1:** Peach *PG* genes identified in this study.

Gene Name	Gene ID	Description	Deduced Polypeptide			Signal Peptide	Domains
			Length (aa)	*M*_W_ (kDa)	PI		
*PpPG1*	ppa018113m	Polygalacturonase At1g48100	510	54.48	6.7	+	I, II, III, IV
*PpPG2*	ppa005391m	Polygalacturonase At1g48100	463	50.16	4.88	−	I, II, III, IV
*PpPG3*	ppa026115m	Exopolygalacturonase clone GBGE184	376	40.07	8.84	−	I, II, III, IV
*PpPG4*	ppa015208m	Exopolygalacturonase clone GBGE184	376	40.06	8.74	−	I, II, III, IV
*PpPG5*	ppa1027214m	-	470	51.15	9.53	+	I, II, III, IV
*PpPG6*	ppa004002m	Polygalacturonase At1g48100	536	57.83	8.8	+	I, II, III, IV
*PpPG7*	ppa015822m	Probable polygalacturonase At3g15720	379	40.23	7.23	−	I, II, III, IV
*PpPG8*	ppa015678m	Probable polygalacturonase At3g15720	390	41.35	6.35	+	I, II, III, IV
*PpPG9*	ppa017135m	Polygalacturonase	292	30.67	6.28	−	I, II, III
*PpPG10*	ppa026655m	Polygalacturonase	375	40.83	9.09	−	I, II, III, IV
*PpPG11*	ppa018224m	Probable polygalacturonase	463	50.68	6.56	+	I, II, IV
*PpPG12*	ppa020086m	Probable polygalacturonase	517	55.99	8.91	+	I, II, IV
*PpPG13*	ppa005310m	Probable polygalacturonase	467	51.58	5.74	+	I, II, IV
*PpPG14*	ppa014982m	Probable polygalacturonase At1g80170	449	48.85	8.74	+	I, II, III, IV
*PpPG15*	ppa018901m	Probable polygalacturonase	391	42.72	6.66	−	I, II, IV
*PpPG16*	ppa005185m	Probable polygalacturonase	473	52.28	8.73	+	I, II, IV
*PpPG17*	ppa024649m	Polygalacturonase At1g48100	437	47.60	5.95	−	I, II, III, IV
*PpPG18*	ppa021095m	Probable polygalacturonase At3g15720	621	65.91	6	+	I, II, III, IV
*PpPG19*	ppa021953m	Polygalacturonase	376	39.46	8.35	−	I, II, III, IV
*PpPG20*	ppa025787m	Polygalacturonase	392	40.98	9.28	+	I, II, III, IV
*PpPG21*	ppa006839m	Polygalacturonase	393	41.33	6.24	+	I, II, III, IV
*PpPG22*	ppa006857m	Polygalacturonase	393	41.26	6.24	+	I, II, III, IV
*PpPG23*	ppa005015m	Probable polygalacturonase	481	52.39	6.36	+	I, II, IV
*PpPG24*	ppa004996m	Probable polygalacturonase	482	52.45	5.81	+	I, II, IV
*PpPG25*	ppa005818m	Probable polygalacturonase	442	48.32	7.12	+	I, II, IV
*PpPG26*	ppa019727m	Probable polygalacturonase At3g15720	382	41.13	8.66	−	I, II, III, IV
*PpPG27*	ppa025098m	Polygalacturonase	384	40.72	7.54	−	I, II, III, IV
*PpPG28*	ppa014719m	Polygalacturonase At1g48100	419	45.94	8.27	−	I, II, III, IV
*PpPG29*	ppa025464m	Polygalacturonase	381	40.56	8.76	−	I, II, III, IV
*PpPG30*	ppa016722m	Polygalacturonase	432	46.30	8.72	−	I, II, III, IV
*PpPG31*	ppa018682m	Polygalacturonase	371	39.42	8.17	−	I, II, III, IV
*PpPG32*	ppa018149m	Polygalacturonase	307	33.04	8.18	−	I, II, III
*PpPG33*	ppa018308m	Polygalacturonase	376	39.92	7.89	−	I, II, III, IV
*PpPG34*	ppa023489m	Probable polygalacturonase At3g15720	436	46.94	7.84	+	I, II, III, IV
*PpPG35*	ppa022427m	Polygalacturonase	431	46.44	5.81	−	I, II, III, IV
*PpPG36*	ppa018519m	Probable polygalacturonase At3g15720	405	43.79	5.82	+	I, II, III, IV
*PpPG37*	ppa005960m	Probable polygalacturonase	435	47.64	5.75	+	I, II
*PpPG38*	ppa004793m	Probable polygalacturonase	491	55.03	9.15	−	I, II, IV
*PpPG39*	ppa021427m	Probable polygalacturonase At3g15720	383	41.32	7.92	−	I, II, III, IV
*PpPG40*	ppa007271m	Polygalacturonase	375	38.92	8.51	−	I, II, III, IV
*PpPG41*	ppa020072m	Polygalacturonase	395	42.03	9.52	+	I, II, III, IV
*PpPG42*	ppa016502m	Polygalacturonase	403	42.82	9.24	+	I, II, III, IV
*PpPG43*	ppa019563m	Exopolygalacturonase	387	41.55	8.93	−	I, II, III, IV
*PpPG44*	ppb019654m	Polygalacturonase	382	30.80	8.81	+	I, IV
*PpPG45*	ppa015568m	Exopolygalacturonase clone GBGE184	482	50.57	6.32	+	I, II, III, IV

## Supplementary Materials

Supplementary materials can be found at www.mdpi.com/1422-0067/17/11/1933/s1.
