# Supplementary material for: Identification and Expression Analysis of Polygalacturonase Family Members during Peach Fruit Softening
Source: Int J Mol Sci. 2016 Nov 18;17(11):1933. doi: 10.3390/ijms17111933 (PMC5133928; doi:10.3390/ijms17111933)
Supplement: Supplementary file 1 [file ijms-17-01933-s001.pdf]

Supplementary Materials: Identification and Expression Analysis of Polygalacturonase Family Members during Peach Fruit Softening

Ming Qian, Yike Zhang, Xiangyan Yan, Mingyu Han, Jinjin Li, Fang Li, Furui Li, Dong Zhang and Caiping Zhao

Table S1. The standardized FPKM values of 16 PpPG genes in RNA-seq of QJB fruit.

Name	Postharvest Days (d)		
	**0**	2	4
PpPG1	1.5	2	17.5
PpPG6	31.5	38	27
PpPG10	0	3	24.5
PpPG11	34.5	18	9
PpPG12	7.5	170	1235
PpPG13	133.5	364	293.5
PpPG14	423.5	416	247.5
PpPG15	1	10	53.5
PpPG21	6	2896	30,400
PpPG22	2.5	1435	15,699
PpPG23	71	205.5	312.5
PpPG24	695.5	933	858
PpPG25	751.5	898	1193
PpPG35	18	8.5	16
PpPG38	1017	963.5	874.5
PpPG39	1.5	0.5	0.5

Table S2. Sequences of the primers used for quantitative reverse transcription PCR analysis.

Gene Name	GenBank Accession No.	Primers for qRT-PCR (5′–3′)
PpPG1	ppa018113m	F: GTGTTGGACTATGGTGCTA<br>R: AGATTGGTTTGACGAGGAA
PpPG6	ppa004002m	F: ATAGTGGTGGTATCAGTGGAT<br>R: GCTTGGTGGAGTGTCATC
PpPG10	ppa026655m	F: CAAATCTCCTATCACCCTCAA<br>R: GCCACCATATCCTTCCATT
PpPG11	ppa018224m	F: GGAGGAGTTGGAGATGGAAAGACA<br>R: GAGAGTGAAGTGGCTGATGAGGTT
PpPG12	ppa020086m	F: TCCTAAGAGCCGCAGAATGTAGAAC<br>R: AGATGATGAATGGCCGCCTTGAA
PpPG13	ppa005310m	F: CTCATCAGCCATCTAACCTT<br>R: ATGTCTTCCACCTATCAACTC
PpPG14	ppa014982m	F: CGGATGCTTGGATTGGTT<br>R: GGATTTGTTGAGTTGGTCTTG
PpPG15	ppa018901m	F: TTCCAGTCCTGCCTTCAT<br>R: CGATAGTGCCGTTGTTACC
PpPG21	ppa006839m	F: GTCATCTGGTGTCACAATCCTCAACTC<br>R: GTCCACAAGCAACGCCTTCTATCC
PpPG22	ppa006857m	F: CAGCCAAATGTTCCATATAGTCATCAACGA<br>R: CCACAAGCAACGCCTTCTATCCAC
PpPG23	ppa005015m	F: TCTCTGCTCCTCCTCTTCACCATCA<br>R: AACTGCCCGTCAACCATCTCCC
PpPG24	ppa004996m	F: CTGAGATGGTGATGGCAATAG<br>R: ATGGCTGGTGAGGTTGAA
PpPG25	ppa005818m	F: GGTAACTTGCTCTGGGATAG<br>R: ATGTGGCTTGTGAGATTGA
PpPG35	ppa022427m	F: TTACAAGACATCCCACTCAAC<br>R: GAAGTTACCGCCGTCATC
PpPG38	ppa004793m	F: TTGCTGAAGATGCTGAGA<br>R: CCCTGTCCGTTTATGGTA
PpPG39	ppa021427m	F: CGTTCCACCGAAGAGATT<br>R: GAGACCGCTTACACCATT
